# Special Thai Oolong Tea: Chemical Profile and *In Vitro* Antidiabetic Activities

**DOI:** 10.3389/fphar.2022.797032

**Published:** 2022-03-07

**Authors:** Narawadee Rujanapun, Wuttichai Jaidee, Thidarat Duangyod, Pravaree Phuneerub, Napassawan Paojumroom, Tharakorn Maneerat, Chuchawal Pringpuangkeo, Salfarina Ramli, Rawiwan Charoensup

**Affiliations:** ^1^ Medicinal Plant Innovation Center of Mae Fah Luang University, Mae Fah Luang University, Chiang Rai, Thailand; ^2^ School of Integrative Medicine, Mae Fah Luang University, Chiang Rai, Thailand; ^3^ Center of Chemical Innovation for Sustainability, School of Science, Mae Fah Luang University, Chiang Rai, Thailand; ^4^ Doi Chang Tea Co., Ltd., Mae Lao, Thailand; ^5^ Faculty of Pharmacy, Universiti Teknologi MARA Cawangan Selangor, Selangor, Malaysia; ^6^ Interactive Pharmacogenomics Institute (iPROMISE), Universiti Teknologi MARA Cawangan Selangor, Selangor, Malaysia

**Keywords:** peaceful rest tea, eternity tea, oolong teas, hyperglycemia, Thai botanical drug, antidiabetic

## Abstract

Special Thai oolong tea is oolong tea (*Camellia sinensis* (L.) Kuntze) steamed with selected Thai botanical drugs. Oolong tea steamed with ginger (*Zingiber officinale*), lemongrass (*Cymbopogon citratus*), and celery (*Anathallis graveolens* L.) is called eternity tea (EN), whereas peaceful rest (PR) tea is made of oolong tea leaves steamed with Indian gooseberry (*Phyllanthus emblica*), Turkey berry (*Solanum torvum*), and wild betel leaf bush leaves (*Piper sarmentosum*). Oolong tea is known for its numerous biological activities including antidiabetic properties. However, the effect of the additional botanical drugs on the biological activities of special oolong teas has not yet been explored. From the results, the PR extract exhibited the best activity in the *in vitro* assays relevant to antidiabetic properties such as chemical antioxidant, anti-inflammation, anti-adipogenesis, enzyme inhibition, and glucose uptake and consumption. The UHPLC-QTOF-MS/MS profiles of PR and EN extracts indicated chemical profiles different from oolong tea. For instance, gingerdiol and gingerol were detected in EN, whereas piperettine I was detected in PR. Therefore, it was inferred that among the three tea extracts, the additional compounds in PR contributed to good activities compared to oolong and EN. It is also important to highlight that the PR extract inhibited glucose uptake and consumption by adipocytes and skeletal muscles at concentrations of 500 and 100 μg/ml, respectively, as well as metformin activity (*p* < 0.05). Findings from this study support the antidiabetic potential of PR tea.

## Introduction

Globally, the incidence of unhealthy lifestyle-derived diseases such as type 2 diabetes (T2D) and cardiovascular diseases has increased yearly. Uncontrolled hyperglycemia is a major cause of blindness, kidney failure, and lower limb amputation, whereas the unnecessary expansion of adipose tissue causes metabolic disorders such as insulin resistance, dyslipidemia, and hypertension. Without proper interventions, it is anticipated that this situation may lead to the growing death rate and health burden due to diabetes in the year 2025 (Lin et al., 2020). T2D is a complex multifactorial disease resulting from the imbalance of sugar homeostasis. High blood sugar or hyperglycemia could result from insulin resistance or pancreatic cells not producing enough insulin to accommodate the sugar level in the blood. Consequently, the impaired glucose homeostasis led to free radical production and inflammation. Pathophysiological pathways associated with oxidative stress and inflammation in diabetes mellitus indicate the progress of the disease (Lima et al., 2021).

Regular exercise and healthy diet have been suggested as able to control the progression of diabetes and cardiovascular diseases. Furthermore, regular intake of beverages such as cocoa, wine, and tea confers health-protective effects due to their antihyperglycemic, enzyme-inhibiting, antioxidant, and anti-inflammatory properties (Martin et al., 2017; Sun et al., 2020).

The intake of oolong [*Camellia sinensis* (L.) Kuntze] tea is popular for preventing obesity and improving lipid metabolism, as demonstrated by their ability to lower total cholesterol and plasma triglyceride levels, along with the reduction in blood pressure and platelet aggregation activity (He et al., 2013). Other reported pharmacological activities of oolong tea and its bioactive compound theasinensins are anticancer, antioxidant, and antimicrobial activities (Zhang et al., 2019).

Infusing medicinal plants such as ginger (*Zingiber officinale* Roscoe) or cloves (*Syzygium aromaticum* (L.) Merr. and L.M.Perry) in tea has been a beneficial common practice. For instance, green tea extracts enriched with natural components like mint, cloves, and ginger in combination improved the efficacy level of treating oral infections (Somasundaram et al., 2019).

Previously, a study reported the antioxidant and anti-glycation activities of 15 Thai botanical drug teas, including lemongrass (*Cymbopogon citratus* (DC.) Stapf), mulberry (*Morus alba* L.), and betel leaves (*Piper sarmentosum* Roxb.). However, their antioxidants and anti-glycation activities were low compared to green tea, oolong tea, and black tea (Deetae et al., 2012). Nevertheless, it is intriguing to combine tea and Thai botanical drugs into one special tea and explore the biological activities.

In this study, “special Thai oolong tea” is an oolong tea steamed with select Thai botanical drug water extracts. Eternity tea (EN) is made of dried oolong tea leaves steamed with ginger (*Zingiber officinale* Roscoe), lemongrass (*Cymbopogon citratus* (DC.) Stapf), and celery (*Anathallis graveolens* (Pabst) F. Barros) water extracts, while peaceful rest tea (PR) is made of oolong tea leaves steamed with Indian gooseberry (*Phyllanthus emblica* L.), Turkey berry (*Solanum torvum* Sw.), and wild betel leaf bush (*Piper sarmentosum* Roxb.) leaf water extracts.

The aim of the study was to investigate the antidiabetic properties of EN and PR with oolong tea using *in vitro* assays. The total phenolic content and chemical antioxidant assays were also carried out. Additionally, the chemical profiles of EN, PR, and oolong tea extracts were analyzed using the UHPLC-QTOF-MS to investigate the presence of phytochemicals from the botanical drugs in the special oolong tea extracts.

## Materials and Methods

### Plant Sample Material

All tea products, including eternity tea (EN), peaceful rest tea (PR), and oolong tea, were obtained from the Doi Chang organic tea plantation in Chiang Rai, Thailand. EN tea was prepared by steaming dried oolong tea leaves (2 kg) with ginger (2 kg), lemongrass (2 kg), and celery (0.4 kg) for 30 min, whereas PR tea comprises oolong tea leaves (5 kg) steamed with Indian gooseberry (2 kg), turkey berry (1 kg), and wild betel leaf bush leaves (0.4 kg) for 30 min. Next, streamed tea was incubated at 80°C for 2 h. Voucher specimens of these samples were authenticated by Charoensup R and Pringpuangkeo C. and deposited at the Medicinal Plant Innovation Center of Mae Fah Luang University, Thailand.

### Preparation of Water Extract

Each tea sample was extracted with water at 95°C for 30 min and freeze-dried to provide water extracts.

### Inhibition of α-Glucosidase and α-Amylase Enzymes

Tea extracts were determined at concentrations of 0.04, 0.2, 2, 4, and 12 μg/ml. Fifty microliters of tea extracts were mixed with 100 µL of α-glucosidase (0.35 U/mL) (CAS: 9001-42-7) and incubated at 37°C. After 10 min, 100 µL of *p*-NPG (1.5 mM) (CAS: 2492-87-7) was added to the mixture and incubated at 37°C for 20 min. The reaction was terminated by the addition of 1,000 µL of Na_2_CO_3_. Two hundred microliters of the mixture was measured at 405 nm on a microplate reader. Acarbose (CAS: 56180-94-0) was used as a positive control for the α-glucosidase inhibitor. The IC_50_ value was calculated (Striegel et al., 2015). For α-amylase inhibition activity, tea extract concentrations of 0.027, 0.055, 0.27, 0.55, 2.77, and 27.77 μg/ml were tested. Acarbose was used as a positive control. Sixty microliters of tea extract/control solution was mixed with 120 µL of starch solution and incubated at 37°C for 10 min. Then, 180 µL of α-amylase (1 U/mL) (EC no: 232-588-1) was added to the solution and incubated for 30 min. The reaction was terminated by adding 240 µL of 0.1 M HCl (CAS: 7647-01-0), and then 300 µL of 0.1 mM iodine solution (CAS: 7553-56-2) was added. The solution was mixed with 500 µL of 0.2 M acetate buffer (CAS: 126-96-5), and the absorbance was measured using a spectrophotometer. The IC_50_ value was calculated (Kusano et al., 2011).

### Glucose Consumption by Adipocytes and Skeletal Muscle Assay

The Zhang method was used for glucose consumption measurement. The 3T3-L1 preadipocyte cell (ATcc® cL-173™) density was adjusted to a concentration of 1 × 10^5^ cells/mL, and cells were spread onto 96-well microtiter plates (100 μL per well). The cells were cultured with a serial concentration of extracts including 25, 50, 100, 250, and 500 μg/ml for 24 h. Insulin (CAS: 11061-68-0) and metformin (CAS: 1115-70-4) were used as positive controls at concentrations of 1 and 755 µM, respectively. At the end of incubation, 10 μL of suspension or glucose (CAS: 50-99-7) standard medium (0‒1000 mg/L) was measured for the glucose oxidase–peroxidase (GOD-POD) assay (Zhang et al., 2008). For sugar uptake by skeletal muscle cells, the experiments were initiated on day 7 when the differentiation of L6 myoblast cells (ATcc® CRL-1458™) to a myotube was completed. Next, cells were incubated with tea extracts or metformin for 24 h. Cells were washed with Krebs–Ringer bicarbonate buffer twice, incubated with Krebs–Ringer bicarbonate buffer for 1 h, and starved in serum-free PBS containing 0.2% c for 1 h. After incubation, the cells were incubated with 2-NBDG (CAS: 186689-07-6) for 20 min minutes. The samples demonstrated fluorescence intensity at an excitation/emission of 485/530 nm (Yamamoto et al., 2015).

### Anti-Adipogenesis Assay

The 3T3-L1 preadipocyte cell lines were treated with 50 μg/ml tea extracts and metformin, which were cultured in DMEM supplemented with 1 μM dexamethasone (CAS: 50-02-2), 10 μg/ml insulin, and 0.5 mM 3-isobutyl-1-methylxanthine (IBMX) (CAS: 28822-58-4), for 2 days. After 2 days, the medium was changed to DMEM containing 10 μg/ml insulin for 2 days. At the end of incubation, the medium was changed to DMEM until day 10. Lipid accumulation was assessed using oil red O staining (CAS: 1320-06-5). The samples were observed and recorded under a microscope. After that, the cells were dissolved in DMSO (CAS: 67-68-5), and the absorbance was measured at 510 nm (Cheng et al., 2006; Bunkrongcheap et al., 2014).

### Viability Assay Using RAW 264.7 and K562 Cells

The cytotoxicity was determined with MTT assay. RAW 264.7 (ATcc® TIB-71™) cells and K562 lymphoblastoid human erythroleukemia cell line (ATcc® CRL-3343™) were seeded at 4 × 10^4^ cells/well in 96-well plates were treated with different concentrations of tea extracts (6.25, 12.5, 25, 50, 100, 250, and 500 μg/ml), for 24 h. Next, the cells were washed with PBS and incubated with 0.5 mM MTT (CAS: 57360-69-7) reagent and 0.15 mg/ml resazurin (CAS: 62758-13-8), respectively, for 4 h. The detection of viable cells was performed with a microplate reader (Fares et al., 2015; Nam et al., 2017).

### Anti-Inflammatory Assay

RAW 264.7 cells were seeded at 4 × 10^4^ cells/well in 96-well plates, incubated with 1 μg/ml LPS (EC no. 297-473-0) for 1 h, and treated with various concentrations of tea extract, including 6.25, 12.5, 25, 50, and 100 μg/ml, for 24 h. NO production was measured using Griess reagent (EC no. 215-981-2). The data were presented as the IC_50_ (Kang et al., 2016).

### Chemical Antioxidant Assays

#### DPPH Free Radical Scavenging Activity

The three replications of tea extracts were tested at concentrations of 0.25, 0.5, 1.25, and 2.5 μg/ml. Five hundred microliters of tea extract solution was mixed with 500 µL of 59 µM DPPH solution (CAS 1898-66-4) in methanol (CAS 67-56-1). (+)-Catechin hydrate (CAS No- 154-23-4) was used as a positive control, and triplicate measurements were carried out. Next, the IC_50_ was calculated (Duangyod et al., 2015).

#### ABTS Free Radical Scavenging Activity

The photometric assay was conducted on a mixture of 500 µL of ABTS solution (CAS:30931-67-0) and 500 µL of tea extract (0.5, 1.25, 2.5, 3.5, and 5 μg/ml). (+)-Catechin hydrate was used as a positive control, and triplicate measurements were carried out. The percentage of scavenging activity and IC_50_ was calculated (Duangyod et al., 2015).

#### Total Phenolic Content

The total phenolic content was determined following the Duangyod study. In total, 800 µL of sample extracts at 1 mg/ml concentration and 200 µL of 15% Folin–Ciocalteu reagent (CAS:12,111-13-6) were added to the test tube, and the volume was adjusted to 2.0 ml with water. The mixture was left for 5 mins. Next, 1.0 ml of Na_2_CO_3_ (0.106 g/ml) (CAS: 497-19-8) was added. The mixture was kept in the dark at room temperature for 60 min. The absorbance was measured at 756 nm. The total phenolic content of the tea extracts was determined through a linear gallic acid standard curve and expressed as milligrams of gallic acid equivalents (GAE) (CAS: 497-19-7) per gram of dry extract (Duangyod et al., 2015).

### UltraHigh-Performance Liquid Chromatography Quadrupole Time-of-Flight Mass Spectrometry Analysis

An Agilent 6500 Series LC Q-TOF System with a column (C-18 2.1*50 mm, 1.7 µm) from Zorbax Eclipse Plus was utilized. The mobile phase was composed of solvent A: H_2_O + 0.1% formic acid and solvent B: ACN +0.1% formic acid. The flow rate was 400 μL/min. The elution was performed starting at 5% B, which was linearly increased to 95% B in 1 min, after which a linear gradient was applied to 17% B in 13 min and 100% B in 22 min. The injection volume was 1.0 μL for the measurements in both positive and negative modes. The instrument parameters were a gas temperature of 350°C, a gas flow of 13 L/min, and a nebulizer pressure of 45 psig. Agilent Mass Hunter Qualitative Analysis Software, version 8.00, was used for the initial processing of the LC/MS data. Compounds were revealed using the Molecular Feature Extractor (MFE) tool in the software. Mass Hunter Profiler Professional (MPP), version 15.1, was used for statistical analysis to profile the samples, such as principal component analysis (PCA). The identification of the peaks was made based on retention time, the analysis of precursor ions, adduct, and error.

### Statistical Analysis

Statistical analysis was performed using SPSS 12.0 software for Windows. Means between the control and treated groups were compared and analyzed using one-way analysis of variance (ANOVA), followed by Tukey’s post hoc test. The value of *p* < 0.05 was considered statistically significant.

## Results and Discussion

In this study, the chemical profiles and antidiabetic properties of oolong tea and special oolong teas, PR and EN, were evaluated by *in vitro* assays. The properties of the tea extracts were compared with the activities of antidiabetic drugs; acarbose, metformin, and insulin. Hyperglycemia is a serious problem for diabetic patients; therefore, inhibiting α-amylase and α-glucosidase enzymes is useful for the control of hyperglycemia. By inhibiting the enzymes from hydrolyzing carbohydrates to simple sugar, the absorption of glucose after food intake can be delayed. From the results, all tea extracts showed α-amylase and α-glucosidase inhibition activity (Table 1). The IC_50_ of the extracts was better than that of acarbose. A lower IC_50_ for α-amylase inhibition than α-glucosidase by the tea extracts was also noted.

**TABLE 1:** α-Glucosidase and α-amylase inhibition assay.

Tea extract	IC_50_ (µg/ml)	
	α-Glucosidase	α-Amylase
PR	1.22 ± 0.05^d^	<0.03^c^
EN	3.50 ± 0.03^b^	1.73 ± 0.23^b^
Oolong	1.48 ± 0.004^c^	<0.03^c^
Acarbose	75.71 ± 0.34^a^	28.73 ± 1.28^a^

Data are represented as mean ± standard deviation (SD). *p* ≤ 0.05 is statistically significant. PR, peaceful rest tea; EN, eternity tea.

**FIGURE 1:**
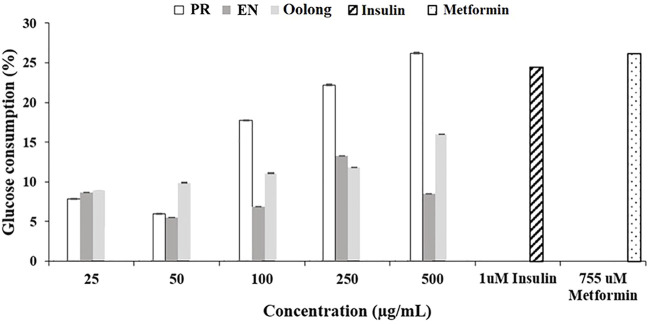
Effect of tea water extracts on glucose consumption in 3T3-L1 cells. PR, peaceful rest tea; EN, eternity tea.

**FIGURE 2:**
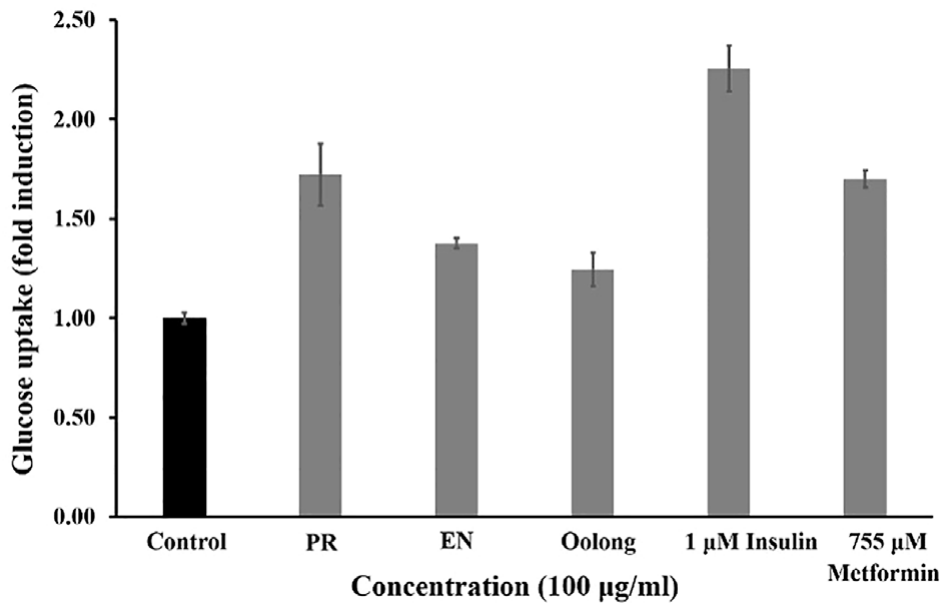
Effect of PR, EN, and oolong tea water extracts at concentration of 100 μg/ml on glucose uptake in L6 cells. PR, peaceful rest tea; EN, eternity tea.

Insulin binding to its receptors enhances glucose transport to skeletal muscle and adipose tissue, which is important for preventing hyperglycemia. Generally, the more glucose is uptaken and consumed by the cells, the less sugar will be available in the blood. As observed in Figure 1, PR extracts affected the cells’ glucose consumption in a dose-dependent manner (50, 100, 250, and 500 μg/ml), but such pattern was not observed in oolong tea and EN extracts. Treatment with insulin resulted in about 25% glucose consumption, whereas treatment with 500 μg/ml PR extracts showed the greatest enhancement of glucose consumption by 3T3-L1 preadipocyte cells. It was noted that the glucose consumption activity of 500 μg/ml PR was not significantly different from that of 755 uM metformin (*p* > 0.05). Next, all tea extracts enhanced glucose uptake in L6 cells compared to the control at a concentration of 100 μg/ml (Figure 2). The glucose uptake was the highest in the L6 cells treated with PR extract, and the activity was not significantly different from 755 uM metformin (*p* > 0.05).

**FIGURE 3:**
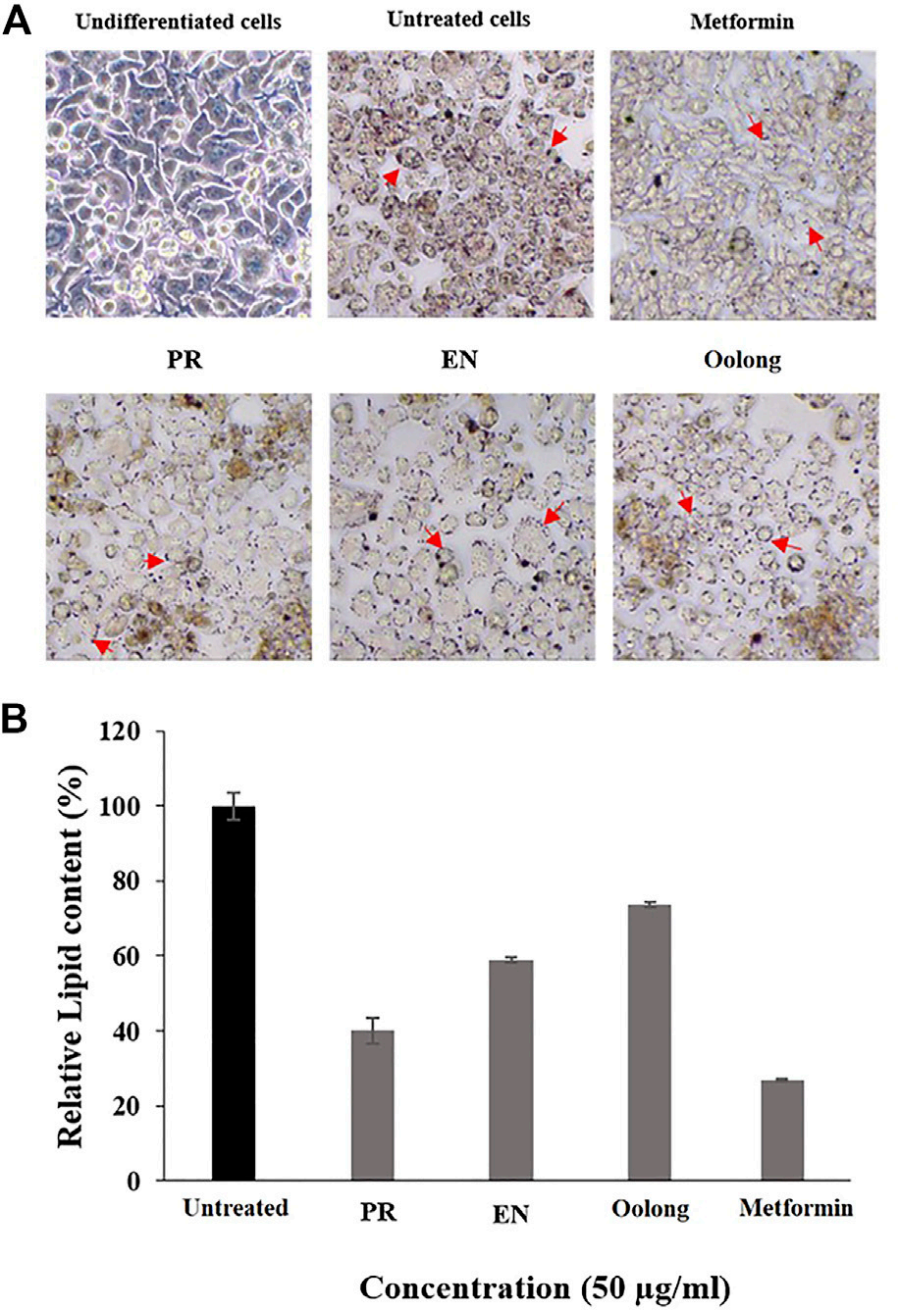
Effect of tea water extracts (50 μg/ml) on intracellular lipid accumulation. **(A)** Lipid droplets of adipocyte cells after red oil O staining. **(B)** Percentage of relative lipid content in adipocyte cells after tea water treatment. Arrows indicate lipid droplets. PR, peaceful rest tea; EN, eternity tea.

The inhibition of lipid accumulation in cells is essential to prevent insulin resistance in diabetic patients. From the results, the intracellular lipid accumulation in adipocyte cells was reduced by all tea extracts, especially PR extracts, where the least lipid droplets were observed compared to cells treated with EN and oolong tea extracts (Figure 3A). Furthermore, untreated cells showed the highest percentage of relative lipid accumulation (Figure 3B). Metformin induces adipocyte differentiation at low concentrations, while higher concentrations of metformin inhibit adipogenesis *via* AMPK activation (Chen et al., 2018). Although the PR extract exhibited 40% of relative lipid content compared to metformin (Figure 3), the percentage is the lowest among other tea extracts.

Inflammation control is important to managing diabetes complications as inflammation also causes insulin resistance (Yang et al., 2018). Nitric oxide (NO) is one of the pro-inflammatory mediators produced during inflammation. In the non-treated RAW 264.7 cells, the inflammatory response induced by LPS resulted in a 100% production of NO. Cells treated with tea extracts showed a reduction in nitrite accumulation in a concentration-dependent manner. Treatment with 100 ug/ml of tea extracts reduced NO production by 20% (Figure 5B). It was also observed that the anti-inflammatory activity of EN and PR extracts was not significantly different (*p* < 0.05).

**FIGURE 4:**
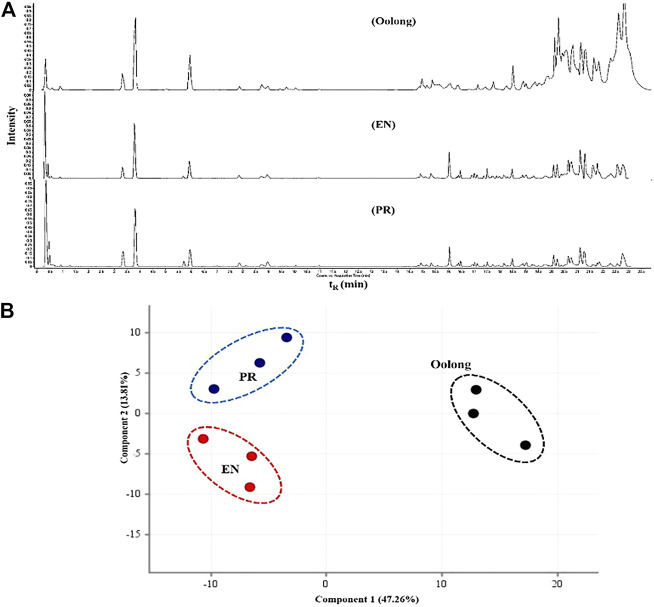
Chemical profile of tea extracts. **(A)** Total ion chromatogram of oolong, EN, and PR. **(B)** Score plot PCA of the chemical compounds from oolong, EN, and PR. RT, retention time; PR, peaceful rest tea; EN, eternity tea.

Next, the chemical profile of each tea extract by LC-MS chromatograms was carried out to rationalize the findings from the *in vitro* assays (Figure 4A). Principal component analysis (PCA) visualized the abundance variations for the metabolites with significant differences, indicating the chemical profiles of each tea are different, *p* < 0.05 (Figure 4B). Chemical analysis showed that compounds detected in the tea extracts comprised the flavonoids, catechin, and phenolic acid (Table 2). This result corroborates the high TPC values and good chemical antioxidant activity exhibited by tea extracts (Table 3). The activity of tea extracts was significantly different from that of catechin hydrate (*p* < 0.05). The generation of reactive oxygen species (ROS) and its association with oxidative stress play a crucial role in the pathogenesis of T2D. Antioxidant results indicated the ability of tea extracts to donate electrons or hydrogen to the DPPH and ABTS free radicals. Both chemical antioxidant assays have been useful for defining the chemical profiles of extracts and quality control.

**TABLE 2:** Antioxidant activities and total phenolic content of tea extracts.

Tea extract	Antioxidant		Total phenolic content
	IC_50_ (µg/ml)		(µg GAE/mg)
	DPPH	ABTS	
PR	1.84 ± 0.02^b^	2.37 ± 0.02^b^	62.07 ± 0.0012^c^
EN	2.48 ± 0.01^d^	3.12 ± 0.04^c^	61.29 ± 0.0026^b^
Oolong	2.77 ± 0.00^c^	3.13 ± 0.00^c^	68.37 ± 0.0021^a^
(+)-Catechin hydrate	0.82 ± 0.04^a^	0.83 ± 0.01^a^	—

Data are represented as mean ± standard deviation (SD). *p* ≤ 0.05 is statistically significant. PR, peaceful rest tea; EN, eternity tea; GAE, gallic acid equivalents.

**TABLE 3:** List of compounds tentatively identified in PR tea, EN tea, and oolong tea by UHPLC-QTOF-MS analysis.

Compound	Formula	*m/z*	Fragment	RT (min)		
				PR	EN	Oolong
Theanine	C_7_H_14_N_2_O_3_	175.1079	140, 125, 104	0.35	—	—
6-Hydroxy-7,4-dimethoxyflavone	C_14_H_17_O_5_	321.1156	175, 104	—	0.35	—
*p*-Coumaric acid	C_9_H_8_O_3_	182.0812	175, 104	—	0.50	—
Gallocatechin	C_15_H_14_O_7_	307.0814	294, 173, 139, 121	3.33	3.34	3.35
Caffeine	C_8_H_10_N_4_O_2_	195.0878	195, 137, 125, 121	3.81	3.81	3.85
Kaempferol 3-*O*-arabinoside	C_20_H_18_O_10_	441.1732	350, 251, 171, 148	4.84	—	—
Gallocatechin gallate	C_22_H_18_O_11_	459.0924	289, 221, 139, 121	—	—	5.95
Epicatechin-3-gallate	C_22_H_18_O_11_	459.0923	289, 221,121	—	5.95	5.98
Myricetin 3-galactoside	C_21_H_20_O_13_	481.2621	446, 350, 251, 150	7.87	—	7.89
Epiafzelechin	C_15_H_14_O_5_	275.1869	193, 185, 136, 125	8.73	8.73	8.73
5-*O*-Caffeoylquinic acid	C_16_H_18_O_9_	355.1727	221, 181, 125	9.68	—	—
Myricitrin	C_21_H_20_O_12_	487.2152	398, 294, 221, 181	—	10.47	—
Luteolin-8-C-glucoside	C_21_H_19_O_11_	448.1009	446, 320, 171	—	—	10.96
Diacetoxy-8-gingerdiol	C_21_H_32_O_6_	381.1894	325, 294, 227	—	14.99	—
Neopellitorine B	C_15_H_25_NO	236.1621	221, 181, 136	15.47	—	—
Theobromine	C_7_H_8_N_4_O_2_	203.1049	136, 110	15.59	—	15.59
8-Gingerol	C_19_H_30_O_4_	323.1468	294, 262, 221, 136	—	16.81	—
Catechin	C_15_H_14_O_6_	291.0864	136, 122	16.86	16.86	16.87
Guanosine	C_10_H_13_N_5_O_5_	284.3312	284, 262, 136, 122	17.71	17.71	17.72
3-Acetoxy-6-gingerdiol	C_19_H_30_O_5_	361.2348	294, 262, 136	—	17.97	—
Piperettine I	C_19_H_21_NO_3_	312.3621	301, 136, 103	18.33	—	—
3-*p*-Coumaroylquinic acid	C_16_H_18_O_8_	361.2347	164, 144, 116	—	—	18.42
6-Gingerdiol	C_17_H_28_O_4_	297.2325	294, 276, 150	—	18.87	—
4-Caffeoylquinic acid	C_16_H_18_O_9_	355.2817	164, 136, 122	—	—	18.97
1-Caffeoylquinic acid	C_16_H_18_O_9_	355.2806	164, 136, 122	—	—	18.97
Kaempferol	C_15_H_10_O_6_	309.2041	262, 237, 221, 185, 150	19.02	19.02	—
3-*O*-methylellagic acid	C_15_H_8_O_8_	317.17869	315, 262, 229	19.22	—	—
Epicatechin gallate	C_22_H_18_O_10_	443.3343	294, 150, 136	19.30	19.29	19.30
Epicatechin 3-*O*-(3-*O*-methyl gallate)	C_23_H_20_O_10_	457.3509	406, 294, 136, 122	—	—	19.80
Theasinensin A	C_44_H_34_O_22_	915.6004	871, 588, 294	—	—	20.314
Isogingerenone B	C_22_H_26_O_6_	387.3752	294, 276, 262, 136	—	20.79	—
Quercetin 3-*O*-glucosylrutinoside	C_33_H_40_O_21_	773.5175	736, 425, 304, 209	21.69	—	21.696
6-*C*-Hexosyl-8-*C*-pentosylluteolin	C_27_H_30_O_16_	581.4343	425, 294, 150	—	21.86	—
Kaempferol 7-*O*-rutinoside	C_27_H_30_O_15_	595.4149	409, 294, 262	22.19	—	22.18
Myricetin 3-(2″-galloylrhamnoside)	C_29_H_26_O_16_	631.1132	610, 549, 294	—	—	22.23

m/z, mass/charge; RT, retention time; PR, peaceful rest tea; EN, eternity tea.

Previous studies on oolong tea reported the anti-obesity and antioxidant properties of the tea (Zhang et al., 2019). Frequently, the polyphenols, particularly the tea catechins, epicatechin, epicatechin-3-gallate, epigallocatechin, and epigallocatechin-3-gallate, have been associated with good antioxidant activity, thus reducing risk of diseases related to oxidative stress (Sanlier et al., 2018). Flavonoid-rich food decreases serum levels of glucose by inducing the insulin-independent 5′ adenosine monophosphate-activated protein kinase (AMPK) pathway. This slows the oxygen consumption of adenosine diphosphate by stimulating GLUT 4 translocation and expression in isolated mitochondria. Such mechanism is similar to that of metformin (Eid et al., 2010). Moreover, molecular studies supported that polyphenols in tea, such as catechins and gallic acid, modulate numerous pathways in the human body, including those involved in antioxidants, inflammation, apoptosis, and lipid metabolism (Shang et al., 2021).

From the chemical profiles of EN and PR, tea catechins are detected in all tea extracts. It is apparent that steaming oolong tea with Thai botanical drugs contributed to additional phytochemicals, especially flavonoids, which could be implied from the findings observed in the *in vitro* assays. For instance, gingerdiol and gingerol detected in EN were previously reported in ginger extracts (Kikuzaki et al., 1992), whereas piperettine I detected in PR was previously reported in *Piper* genus (Parmar et al., 1997). The beneficial effects of both plants on controlling blood sugar have been discussed previously. Ginger supplementation was recommended as effective adjuvant therapy for patients with T2DM (El Gayar et al., 2019), while the hypoglycemic effect of the water extract of *Piper sarmentosum* Roxb. on rats was also reported (Peungvicha et al., 1998).

High-molecular weight phenolic compounds such as theaflavin-3-3′-digallate and thearubigin have been reported to inhibit α-glucosidase (Striegel et al., 2015). However, both compounds were not detected in the tea extracts. However, the presence of quercetin 3-O-glucosylrutinoside was detected in both PR and oolong tea extracts could contribute to the observed α-amylase inhibition activity. The structure–activity relationship of quercetin 3-O-glucosylrutinoside as the α-amylase inhibitor was elucidated in a previous study (Li et al., 2018). The presence of 5-O-caffeoylquinic acid in PR, *p*-coumaric acid in EN, and 4-caffeoylquinic acid and 1-caffeoylquinic acid in oolong tea could contribute to the anti-inflammatory property of the tea extracts. Such compounds were previously observed in the *Cornus officinalis* var. koreana Kitam extract that attenuated the inflammatory response induced by LPS in RAW 264.7 cells (Najjar et al., 2021). Additionally, the anti-inflammatory property of caffeoylquinic acids (CQAs) has also been described (Liu et al., 2020).

**FIGURE 5:**
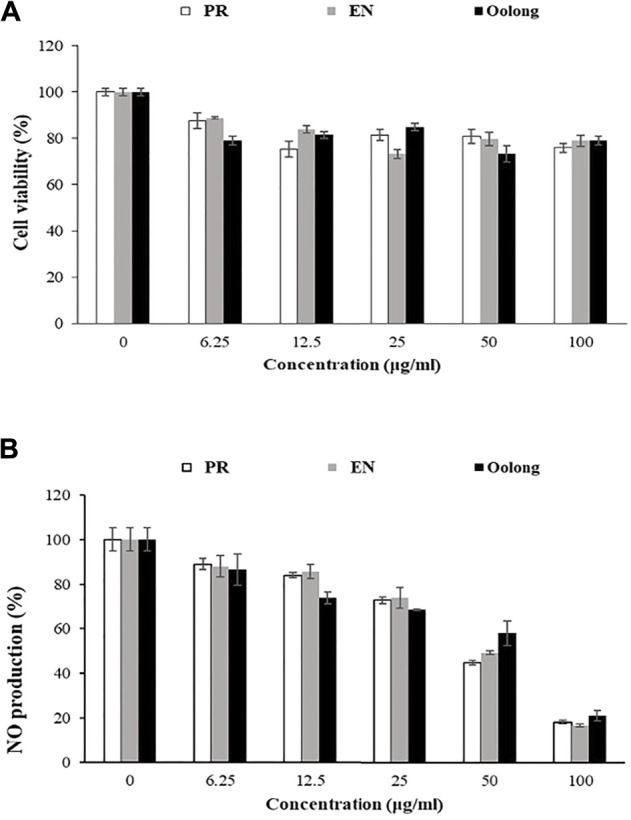
Effect of tea water extracts on RAW 264.7 cells. **(A)** Cell viability percentage. **(B)** Percentage of nitric oxide production in RAW cells after tea extract treatment. PR, peaceful rest tea; EN, eternity tea; NO production, nitric oxide production.

**FIGURE 6:**
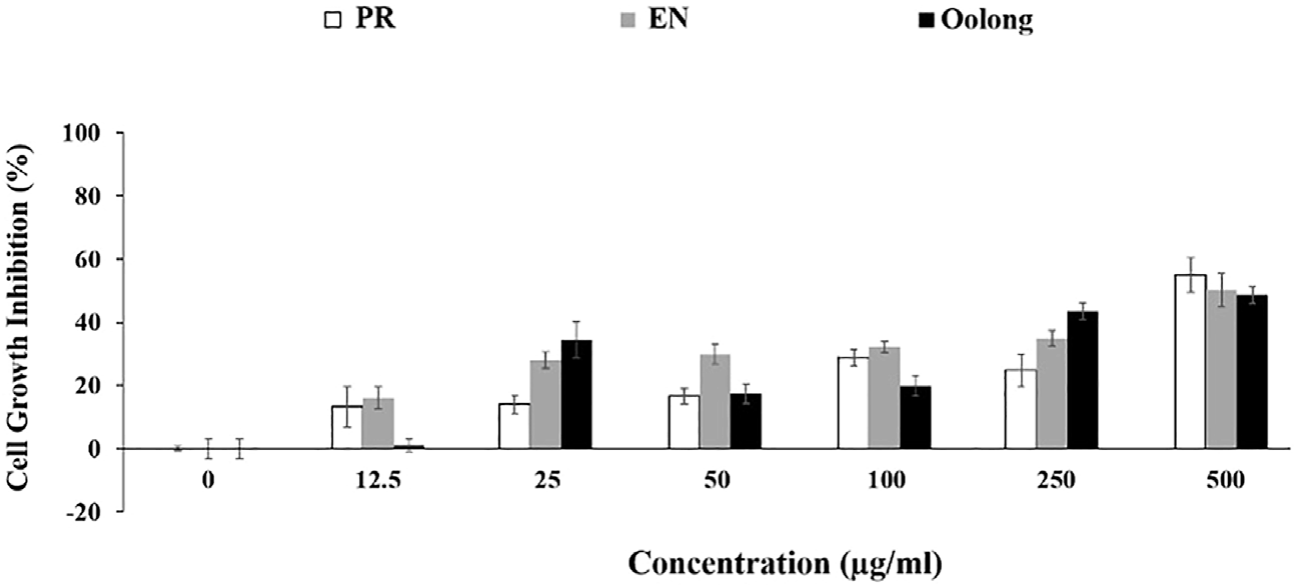
Anti-proliferation effect of tea extracts on K562 cells. PR, peaceful rest tea; EN, eternity tea.

The antiproliferation activity of tea extracts was investigated using K562 and RAW264.7 cells. The results indicated the tea extracts were not toxic to both cells (Figures 5A,6). Evaluating tea quality, efficacy, and safety is important for the tea industry (Lin and Sun, 2020), especially to promote both safe and rational use of these special oolong teas. Finally, from the *in vitro* results and chemical profiles of tea extracts, this study rationalizes the consumption of oolong and special oolong teas. Nevertheless, possible contraindications and interactions when the tea is taken together with pharmaceuticals such as anti-diabetic drugs and functional foods (May and Schindler, 2016) are suggested for future study.

## Conclusion

Oolong tea is already good on its own. Thai botanical drugs are steamed with the oolong tea to produce the special oolong teas, EN and PR. The extra compounds observed in the chemical profiles of PR and EN teas rationalized the good *in vitro* antidiabetic property of the extracts. Interestingly, among all the tea extracts, PR extracts exhibited the best activity in the *in vitro* assays, particularly the inhibition of glucose uptake and consumption by adipocytes and skeletal muscle. Other than tea catechins, the activities could be due to the presence of flavonoid compounds contributed by the Thai botanical drugs. Based on the results, this study suggests the potential of PR extracts to combat diabetes, cardiovascular diseases, and obesity.

## Data Availability

The raw data supporting the conclusion of this article will be made available by the authors, without undue reservation.
