# Supplementary material for: Special Thai Oolong Tea: Chemical Profile and In Vitro Antidiabetic Activities
Source: Front Pharmacol. 2022 Mar 7;13:797032. doi: 10.3389/fphar.2022.797032 (PMC8936575; doi:10.3389/fphar.2022.797032)
Supplement: Supplementary file 1 [file DataSheet1.pdf]

Supplementary Material

Narawadee Rujanapun [1], Wuttichai Jaidee [1], Thidarat Duangyod [1,2], Pravaree Phuneerub [1,2], Napassawan Paojumroom [1], Tharakorn Maneerat [1,3], Salfarina Ramli[4,5], Rawiwan Charoensup [1,2*]

[1]Medicinal Plant Innovation Center of Mae Fah Luang University, Mae Fah Luang University, Chiang Rai 57100, Thailand, [2]School of Integrative Medicine, Mae Fah Luang University, Chiang Rai 57100, Thailand, [3]Center of Chemical Innovation for Sustainability, School of Science, Mae Fah Luang University, Chiang Rai 57100, Thailand, [4]Faculty of Pharmacy, Universiti Teknologi MARA Cawangan Selangor, 42300 Puncak Alam, Selangor, Malaysia, [5]Interactive Pharmacogenomics Institute (iPROMISE), Universiti Teknologi MARA Cawangan Selangor, 42300 Puncak Alam, Selangor, Malaysia

*** Correspondence:**
Corresponding Author
rawiwan.cha@mfu.ac.th

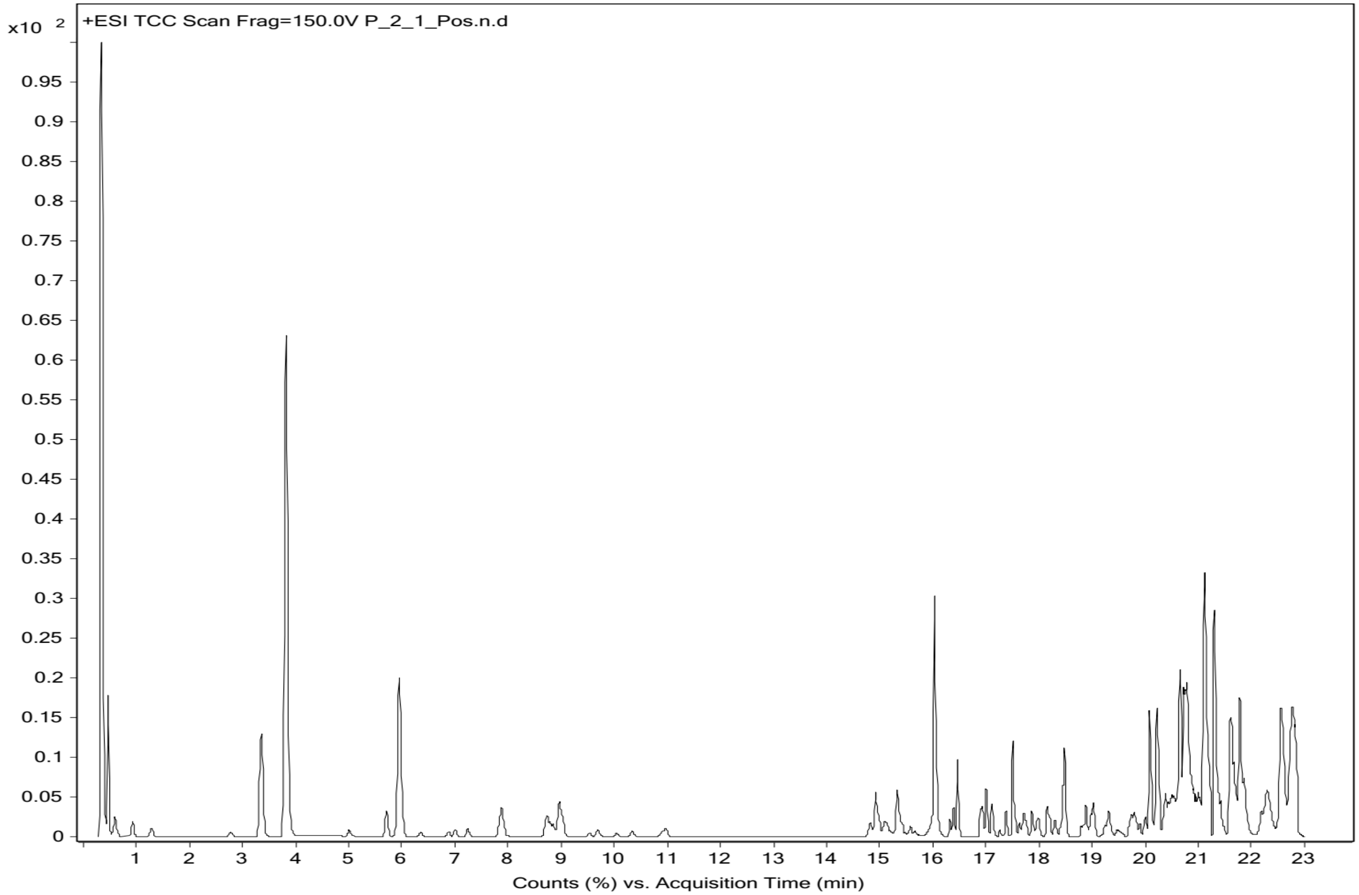

+ESI TCC Scan Frag=150.0V P_2_1_Pos.n.d
x10 2
0.95
0.9
0.85
0.8
0.75
0.7
0.65
0.6
0.55
0.5
0.45
0.4
0.35
0.3
0.25
0.2
0.15
0.1
0.05
0
1 2 3 4 5 6 7 8 9 10 11 12 13 14 15 16 17 18 19 20 21 22 23
Counts (%) vs. Acquisition Time (min)

Supplementary Table S1. Chemical profile of ethanolic PR extract

No.	Identification	t_R (min)	m/z	Mass	Adduct	Formula	Error (ppm)	MS2 fragments	Compound class
1	Choline	0.35	104.1067	103.0996	M+H	C5H15NO	1.74	104, 102	Alkaloids
2	Valine	0.35	140.0685	117.0791	M+Na	C5H11NO2	-1.22	136, 125, 121	Amino acid
3	Theanine	0.35	175.1079	157.0741	M+Na	C7H14N2O3	-1.47	140, 125, 104	Amino acid
4	Caffeine	3.81	195.0879	194.0807	M+H	C8H10N4O2	-1.63	195, 137, 125, 121	Alkaloids
5	Theobromine	15.59	203.1049	180.1149	M+Na	C7H8N4O2	0.6	136, 110	Alkaloids
6	Neopellitorine B	15.47	236.1621	235.1548	M+H	C15H25NO	-0.97	221, 181, 136	Piperidine
7	Glycerophosphocholine	15.14	281.1002	258.1110	M+Na	C8H20NO6P	-2.61	251, 209, 171	Alkaloids
8	Epiafzelechin	8.73	275.1869	274.1796	M+H	C15H14O5	-0.96	193, 185, 136, 125	Catechins
9	Guanosine	17.71	284.3241	283.3241	M+H	C10H13N5O5	-0.72	262, 221, 136, 122	Nucleoside
10	Catechin	5.72	291.0864	290.0791	M+H	C15H14O6	-0.71	136, 122	Catechins
11	Kaempferol	19.02	309.2041	286.2148	M+Na	C15H10O6	-1.65	262, 237, 221, 185, 150	Flavonoids
12	Gallocatechin (GC)	3.33	307.0816	306.0743	M+H	C15H14O7	-0.76	294, 173, 139, 121	Catechins
13	Piperettine I	18.33	312.3621	311.3548	M+H	C19H21NO3	0.99	301, 136, 103	Piperidine
14	*N*-Isobutyl-2,4,10,12-octadecatetraenamide	17.71	332.3312	331.32418	M+H	C22H37NO	0	260, 229	Amide
15	3-*O*-methylellagic acid	19.22	317.17869	316.1713	M+H	C15H8O8	-0.66	315, 262, 229	Phenolic
16	5-*O*-Caffeoylquinic acid	9.68	355.1727	354.1655	M+H	C16H18O9	0.16	221, 181, 125	Phenolic acid
17	Pipgulzarin	17.09	372.3474	371.3400	M+H	C23H33NO3	-0.25	299, 267, 164, 136	Piperidine
18	Benzyl primeveroside	19.97	425.2147	402.2254	M+Na	C18H26O10	-0.82	304, 262, 178, 150	Aroma precursor
19	Kaempferol 3-*O*-arabinoside	4.84	441.1732	418.1840	M+Na	C20H18O10	-0.39	350, 251, 171, 148	Flavonoid glycoside
20	Epicatechin gallate	19.30	443.3343	442.327	M+H	C22H18O10	-3.5	294, 150, 136	Catechins
21	Oleanolic acid	19.86	457.3506	456.3433	M+H	C30H48O3	0.96	413, 369, 229, 178	Fatty acid
22	Epigallocatechin gallate	5.95	459.0922	458.0849	M+H	C22H18O11	-1.1	446, 289, 153, 139	Catechins
23	Myricetin 3-galactoside	7.87	481.2616	480.2544	M+H	C21H20O13	2.7	446, 350, 251, 150	Flavonoid glycoside
24	Kaempferol 7-*O*-rutinoside	22.19	595.4140	594.4073	M+H	C27H30O15	0.04	522, 409, 304, 209	Flavonoid glycoside
25	Quercetin 3-*O*-glucosylrutinoside	21.69	773.5182	772.5112	M+H	C33H40O21	-0.06	736, 425, 304, 209	Flavonoid glycoside

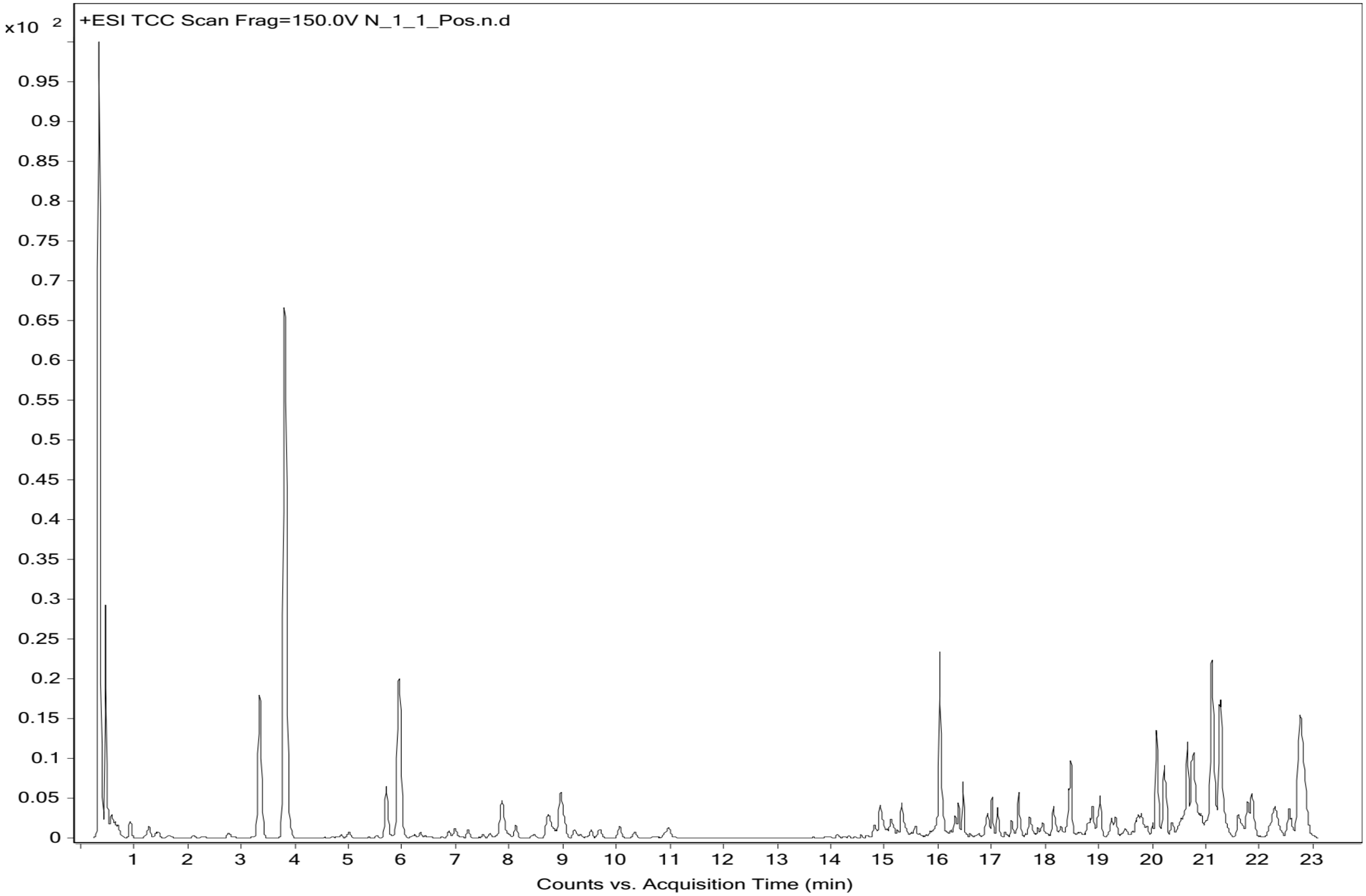

Supplementary Figure S2. Chromatograms of Eternity tea in positive ion mode

Supplementary Table S2. Chemical profile of ethanolic EN extract

No.	Identified Compound	t_R (min)	m/z	Mass	Adduct	Formula	Error (ppm)	MS2 fragments	Compound class
1	Choline	0.34	104.1072	103.0994	M+H	C5H14NO	3.39	104, 102	Alkaloids
2	Betaine	0.35	118.0859	117.0787	M+H	C5H11NO2	2.36	104	Alkaloids
3	Vanillin	5.95	153.0185	152.0112	M+H	C8H8O3	-1.85	139, 125, 121	Amino acid
4	*p*-coumaric acid	0.50	182.0812	164.0486	$M+NH_4$	C9H8O3	-7.57	175, 104	Phenolic acid
5	Caffeine	3.81	195.0879	194.0880	M+H	C8H10N4O2	-1.67	195, 137, 125, 121	Alkaloids
6	Epiafzelechin	8.73	275.1870	274.1797	M+H	C15H14O5	-1.34	193, 185, 136, 125	Catechins
7	Guanosine	17.71	284.3314	283.3241	M+H	C10H13N5O5	-1.01	262, 221, 136, 122	Nucleoside
8	Catechin	16.86	291.2681	290.2607	M+H	C15H14O6	0.75	136, 122	Catechins
9	6-Gingerdiol	18.87	297.2325	296.2254	M+H	C17H28O4	-0.51	294, 276, 150	Gingerol
10	Gallocatechin (GC)	3.34	307.0815	306.0743	M+H	C15H14O7	-0.93	294, 173, 139, 121	Catechins
11	Kaempferol	19.02	309.2043	286.2150	M+Na	C15H10O6	-2.6	294, 262, 164,	Flavonoid
12	6-Hydroxy-7,4′-dimethoxyflavone	0.35	321.1156	298.1264	M+Na	C14H17O5	0.07	175, 104	Flavonoid
13	8-Gingerol	16.81	323.1468	322.1395	M+H	C19H30O4	2.6	294, 262, 221, 136	Gingerol
14	10-Shogaol	9.69	355.1729	332.1837	M+Na	C21H32O3	-0.75	294, 221, 125	Gingerol
15	3-Acetoxy-6-gingerdiol	17.97	361.2348	338.2459	M+Na	C19H30O5	-0.59	294, 262, 136	Gingerol
16	Diacetoxy-6-gingerdiol	14.99	381.1894	380.1821	M+H	C22H34O6	3.39	294, 227,136	Gingerol
17	Isogingerenone B	20.79	387.3752	386.3681	M+H	C22H26O6	-5.31	294, 276, 262, 136	Gingerol
18	Benzyl primeveroside	19.78	425.2879	402.2986	M+Na	C18H26O10	-1.23	304, 262, 178, 150	Aroma precursor
19	Diacetoxy-8-gingerdiol	14.99	381.1894	380.1821	M+H	C21H32O6	3.93	325, 294, 227	Gingerol
20	Epicatechin gallate (ECG)	19.29	443.3341	442.327	M+H	C22H18O10	5.82	294, 262, 150	Catechins
21	Epicatechin-3-gallate	5.95	459.0924	458.0849	M+H	C22H18O11	-0.39	289, 208, 121	Catechins
22	Keampferol 7-galactoside	10.34	471.0901	448.1007	M+Na	C21H20O11	-0.34	431, 294, 221, 125	Flavonoid glycoside
23	Linalool 3,6-oxide primeveroside	10.04	487.2152	464.2258	M+Na	C21H36O11	-0.1	398, 294, 221	Aroma precursor
24	Myricitrin	10.47	487.2152	464.2258	M+Na	C21H20O12	-0.1	398, 294, 221, 181	Flavonoid glycoside
25	6-*C*-Hexosyl-8-*C*-pentosylluteolin	21.86	581.4343	580.4274	M+H	C27H30O16	1.09	425, 294, 150	Flavonoid glycoside

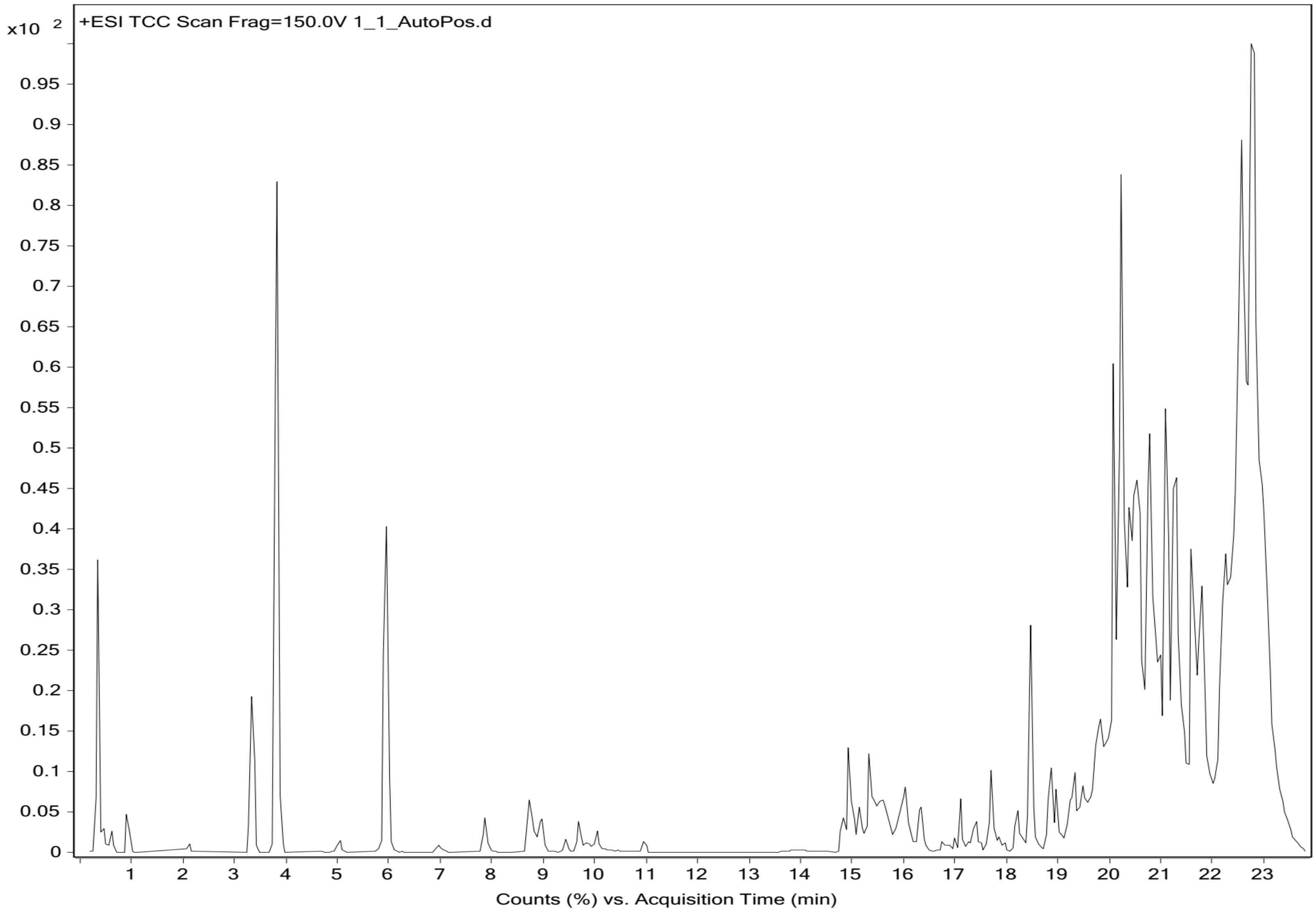

Supplementary Figure S3. Chromatograms of Oolong tea in positive ion mode

Supplementary Table S3. Chemical profile of Oolong tea

No.	Identified Compound	t_R (min)	m/z	Mass	Adduct	Formula	Error (ppm)	MS2 fragments	Compound class
1	Choline	0.32	104.1069	103.0993	M+H	C5H15NO	1.15	104, 102	Alkaloids
2	Betaine	0.35	118.0862	117.0789	M+H	C5H11NO2	0	118, 116, 114, 104	Alkaloids
3	Phenylalanine	0.91	166.0859	165.0712	M+H	C9H11NO2	1.84	166, 125, 121	Amino acid
4	Caffeine	3.85	195.0878	194.0805	M+H	C8H10N4O2	-0.89	195, 137, 125, 121	Alkaloids
5	Theobromine	15.59	203.1049	180.1155	M+Na	C7H8N4O2	-3.98	136, 110	Alkaloids
6	Epiafzelechin	8.73	275.1867	274.1796	M+H	C15H14O5	-0.33	193, 185, 136, 125	Catechins
7	Guanosine	17.72	284.3312	283.3240	M+H	C10H13N5O5	-0.87	284, 262, 136, 122	Nucleoside
8	Catechin	16.87	291.2684	290.2612	M+H	C15H14O6	-0.1	136, 122	Catechins
9	Gallocatechin	3.35	307.0814	306.0742	M+H	C15H14O7	-0.37	294, 173, 139, 121	Catechins
10	Epigallocatechin	17.40	307.1882	306.1809	M+H	C15H14O7	-0.43	294, 221, 136	Catechins
11	3-*p*-Coumaroylquinic acid	18.42	361.2347	338.2455	M+Na	C16H18O8	0.61	164, 144, 116	Phenolic acid
12	4-Caffeoylquinic acid	18.97	355.2817	354.2745	M+H	C16H18O9	-1.85	164, 136, 122	Phenolic acid
13	1-Caffeoylquinic acid	18.97	355.2806	354.2733	M+H	C16H18O9	-1.3	164, 136, 122	Phenolic acid
14	Benzyl primeveroside	22.04	425.3514	402.3622	M+Na	C18H26O10	-3.07	304, 178, 150	Aroma precursor
15	Epicatechin gallate	19.30	443.3345	442.3272	M+H	C22H18O10	-1.24	294, 262, 150	Catechins
16	Luteolin-8-C-glucoside	10.96	448.1009	471.0901	M+Na	C21H19O11	0.07	446, 320, 171	Flavonoid glycoside
17	Epicatechin-3-gallate	5.98	459.0923	458.0851	M+H	C22H18O11	-0.17	289, 221,121	Catechins
18	Epicatechin 3-*O*-(3-*O*-methylgallate)	19.80	457.3509	456.3436	M+H	C23H20O10	0.5	406, 294, 136, 122	Catechins
19	Gallocatechin gallate	5.95	459.0924	458.0850	M+H	C22H18O11	-0.55	289, 221, 139, 121	Catechins
20	Linalool 3,6-oxide primeveroside	10.04	487.2153	464.2260	M+Na	C21H36O11	-0.1	398, 294, 221	Aroma precursor
21	Myricetin 3-galactoside	7.89	481.2621	480.2548	M+H	C21H20O13	1.49	446, 350, 251, 150	Flavonoid glycoside
22	Kaempferol 7-*O*-rutinoside	22.18	595.4149	594.4076	M+H	C27H30O15	-1.6	409, 294, 262	Flavonoid glycoside
23	Myricetin 3-(2''-galloylrhamnoside)	22.23	631.1132	630.1059	M+H	C29H26O16	-3.4	610, 549, 294	Flavonoid glycoside
24	Quercetin 3-*O*-glucosylrutinoside	21.696	773.5175	772.5103	M+H	C33H40O21	-0.36	736, 425, 304, 209	Flavonoid glycoside
25	Theasinensin A	20.314	915.6004	914.5951	M+H	C44H34O22	0.54	871, 588, 294	Dimeric catechin

Supplementary Table S4. DPPH inhibition of tea extracts

Samples	Concentration (μg/ml)	DPPH inhibition (%)	IC50 (μg/ml)
PR	0.25	6.93 ± 1.15	1.84 ± 0.02
	0.5	17.73 ± 1.05	
	1.25	40.00 ± 0.33	
	2.5	67.73 ± 0.68	
	5	111.10 ± 0.63	
EN	0.25	5.56 ± 0.84	2.48 ± 0.01
	0.5	11.65 ± 0.38	
	1.25	29.54 ± 0.77	
	2.5	57.59 ± 0.38	
	5	91.60 ± 0.51	
Oolong	0.25	3.23 ± 0.20	2.77 ± 0.00
	0.5	10.28 ± 0.20	
	1.25	25.60 ± 0.53	
	2.5	50.49 ± 0.20	
	5	85.37 ± 0.53	
(+)-catechin hydrate	0.05	0.37 ± 0.81	0.82 ± 0.04
	0.125	5.97 ± 1.85	
	0.25	16.67 ± 1.84	
	0.5	32.71 ± 1.15	
	1.25	75.99 ± 4.33	

Supplementary Table S5. ABTS inhibition of tea extracts

Samples	Concentration (μg/ml)	ABTS inhibition (%)	IC50 (μg/ml)
PR	0.5	10.30 ± 0.87	2.37 ± 0.02
	1.25	29.77 ± 1.23	
	2.5	54.56 ± 0.31	
	3.5	74.69 ± 0.98	
	5	98.38 ± 0.36	
EN	0.5	5.95 ± 0.26	3.12 ± 0.04
	1.25	19.05 ± 0.85	
	2.5	42.28 ± 0.41	
	3.5	61.46 ± 0.91	
	5	80.20 ± 0.87	
Oolong	0.5	6.68 ± 0.99	3.13 ± 0.00
	1.25	21.16 ± 0.14	
	2.5	42.61 ± 0.57	
	3.5	56.11 ± 0.67	
	5	78.69 ± 0.57	
(+)-catechin hydrate	0.025	7.23 ± 1.09	0.83 ± 0.01
	0.125	11.98 ± 0.21	
	0.25	15.30 ± 2.69	
	0.5	34.30 ± 0.54	
	1.25	72.43 ± 1.10	

Supplementary Table S6. The data of total phenolic content

Samples	Concentration (µg/ml)	Total phenolic content (µg GAE/mg)
PR	1,000	62.22
		61.89
		62.11
EN	1,000	61.44
		61.55
		60.88
Oolong	1,000	68.07
		68.63
		68.40

Supplementary Table S7. α glucosidase inhibition activity of tea extracts

Samples	Concentration (µg/ml)	α-glucosidase inhibition (%)	IC50 (µg/ml)
PR	0.04	1.93 ± 1.67	1.22 ± 0.05
	0.2	4.29 ± 0.74	
	2	63.33 ± 0.63	
	4	86.57 ± 0.23	
	12	95.83 ± 0.00	
EN	0.04	6.02 ± 0.19	3.50 ± 0.03
	0.2	18.32 ± 0.48	
	2	49.05± 0.67	
	4	87.47 ± 0.14	
	12	95.40 ± 0.13	
Oolong	0.04	1.13 ± 0.51	1.48 ± 0.004
	0.2	3.15 ± 0.57	
	2	57.50 ± 0.54	
	4	83.37 ± 0.19	
	12	96.62 ± 0.29	
Acarbose	80	35.34 ± 0.36	75.71 ± 0.34
	120	50.48 ± 0.45	
	200	64.28 ± 0.32	
	320	74.36 ± 0.13	
	400	78.69 ± 0.29	

Supplementary Table S8. α amylase inhibition activity of tea extracts

Samples	Concentration (µg/ml)	α amylase inhibition (%)	IC50 (µg/ml)
PR	0.027	128.95 ± 36.02	< 0.03
	0.055	127.61 ± 0.66	
	0.27	125.34 ± 0.79	
	0.55	119.15 ± 0.35	
	2.77	116.16 ± 0.61	
	5.55	110.61 ± 12.28	
	27.77	106.16 ± 0.20	
EN	0.027	-	1.73 ± 0.23
	0.055	26.10 ± 1.76	
	0.27	31.19 ± 1.38	
	0.55	36.67 ± 4.40	
	2.77	56.27 ± 2.30	
	5.55	79.47 ± 3.09	
	27.77	103.81 ± 2.83	
Oolong	0.027	108.24 ± 6.16	< 0.03
	0.055	139.95 ± 0.96	
	0.27	136.67 ± 1.14	
	0.55	127.71 ± 0.51	
	2.77	123.38 ± 0.87	
	5.55	115.31 ± 17.74	
	27.77	108.92 ± 0.29	
Acarbose	24.03	34.55 ± 0.19	28.73 ± 1.28
	48.07	39.81 ± 0.85	
	96.15	42.88 ± 1.39	
	192.3	47.65 ± 1.14	
	769.23	71.33 ± 2.19	
